# Suggestions about Abdominal and Pelvic Surgery

**Published:** 1891-06

**Authors:** Wm. H. Wathen

**Affiliations:** Louisville, Professor of Abdominal Surgery and Gynecology in the Kentucky School of Medicine, Gynecologist to the Louisville City Hospital, etc.


### SUGGESTIONS ABOUT ABDOMINAL AND PELVIC SURGERY.*

BY WM. H. WATHEN, M. D., OF LOUISVILLE,
Professor of Abdominal Surgery and Gynecology in the Kentucky School of Medicine, Gynecologist to the Louisville City Hospital, etc.

The recent contributions upon abdominal and pelvic surgery are probably more numerous and practical than upon any other department of general or special surgery; still there is a variety of opinion as to the best methods of treating pathological conditions within the peritoneum, or as to the immediate or permanent results of the many procedures that have been practiced.

*Read before the Obstetrical and Gynecological Section of the American Medical Association, May 8, 1891.

This is especially true of pelvic surgery, where we find in the practice of the most experienced and successful operators accidents during the operation, and complications following it, for the prevention of which there is no united opinion as to the correct technique to adopt; nor is it always possible to explain why troublesome complications occur in one case and do not occur in another apparently similar case. Careful observation and experience may finally teach us much wisdom in these matters, and I will ask your kind indulgence while I briefly allude to a few things that may be of value, if carefully discussed by the members.

There is too much laparotomy done, and too many men are doing it; men who know too little about the diagnosis and pathology of abdominal or pelvic diseases, or about the best technique in operating, and have few facilities for doing such work. Continuously good laparotomy work cannot be done except by men who largely devote themselves to this department of special surgery, and with such men some cases are operated on where the indications do not justify it. The appendages are sometimes removed for vague nervous troubles, where there is no disease of the ovaries or tubes, or peritoneal adhesions. Such cases are made worse and are mutilated in a way that cannot be corrected.

The pendulum has swung too far, but many of our best operators are earnestly urging upon the medical profession that the operation is not indicated except in cases where there is well defined disease that has resisted, or will resist, other more conservative means.

As the experience of an honest surgeon widens, he operates relatively, less frequently and he can recall cases that he does not believe should have been operated on. An honest, intelligent, and careful man may, when young in observation and practice, make mistakes in the selection of suitable cases for laparotomy, but this is less frequent than it was a few years ago. It is criminal to do dangerous or capital operations while ignorant of the best methods for doing such work, or for the purpose of adding a little cheap glory to our reputation; or to report cases that apparently recover from the immediate effects of the operation as permanently relieved before the final results can be appreciated. Such men usually have many bad results or deaths that they do not report so promptly, and the profession, or the people, seldom hear much about them.

I have reported but a small minority of my successful cases, but have promptly reported my bad results or deaths because by a careful study of such cases we finally do better work, by learning how to avoid or prevent complications or accidents that may cause the death of our patient. Reported recoveries in simple cases of laparotomy do not always indicate superior or unusual skill in the operator; such reports are of little value to the medical profession and may indirectly result in the death of many women by influencing ignorant men, with no facilities for such work, to attempt it because of its apparent simplicity.

What I may say relative to the technique, etc., of laparotomy refers to cases where the conditions are manifestly such as to positively indicate the necessity for operation. In preparing for an operation, the physical and mental condition, and the hygienic and sanitary surroundings of every patient should be made as perfect as possible under existing circumstances; and unless absolute surgical cleanliness is observed in everything that may come in contact with the wound or peritoneum, septic infection may follow.

Some operators, who talk a good deal about antisepsis, do not know how to be surgically clean, because they have not learned to appreciate the value of cleanliness in every detail before and during the operation. The infection often comes to the patient by the neglect of little things, without the strict observance of which no man can be a successful abdominal surgeon. The danger from atmospheric infection is practically *nil*, as has been shown by Kummel and others, and by the continuously good results in operations done in large amphitheatres before several hundred students. It may be possible for septic matter to reach the peritoneum through the intestinal walls, but this has not been proven. A spray of antiseptic solutions is not necessary, and if strong enough to kill pathogenic germs supposed to be floating in the atmosphere, it is positively poisonous if used during an abdominal section.

Some men, who use the spray Don Quixote-like, while pursuing an imaginary foe, allow the deadly enemy to enter through numerous neglected channels — the hands, sponges, sutures, instruments, etc. Every operator should of course observe the broad principles that make the foundation of all good surgery, but if he neglects the details he will be disappointed in the results. Asepsis is more easily accomplished in well regulated private or public hospitals or infirmaries. In private houses septic matter may be more readily introduced unless the operator, or an experienced nurse, rigorously superintends everything before and during the operation.

That we may better appreciate the practical significance of my position as to what constitutes asepsis in laparotomy, I will give some of the methods before and during an operation. I prefer not to operate in a room where the patient is afterward to stay, and when I am compelled to do so, if delay is admissible, I have the room thoroughly cleansed and ventilated for twenty-four hours before the operation, but use no spray or other means of disinfection. When it can be done I operate in a room at St. Joseph's Infirmary, specially prepared for laparotomy work and so arranged that everything in or about the apartment can be kept aseptic with but little care. The operating tables for the surgeon and nurses have plate glass covers, and the trays for instruments and pans for sponges and dressings are white porcelain-lined.

Everything is carefully cleansed before each operation, and the operator and his chief assistant take a bath and put on clean linen, and white aprons reaching from the neck to below the knees and extending entirely around the body, so as to prevent the hands coming in contact with anything unclean. The towels are carefully washed and boiled and are used for no other purpose. Soft and well shaped sponges, free of sand or grit, are selected, and after being carefully washed are made aseptic after the method of Greig Smith. Eight ounces of bisulphide of soda and four ounces of oxalic acid are dissolved in a gallon of water, in which twelve to twenty sponges are immediately immersed and kept for ten minutes; they are then washed by frequent changes of water for one hour so as to get out all the sulphurous acid and sulphur. This is quite a labor, but it insures perfect freedom of septic matter. They are then wrung out of the water and put into a clean cotton or linen bag so as to keep out the dust while drying. When dry they are put in large ground glass stoppered bottles or jars, and may be kept indefinitely in a pure condition.

Sponges once used may again be made aseptic by the same process, but I prefer not using them a second time if they have been soiled in septic matter. If a sponge comes in contact with anything that may be unclean, it is not used until again prepared. Chinese hard-twist silk of three sizes is used. It is purchased in unbroken packages and wound loosely on separate glass spools. These are put into glass test tubes which are stoppered with a piece of absorbent cotton and then sterilized. They are kept in the sterilizer for an hour, for three consecutive days. The silk is now so free of bacteria that a culture could not be made from it, and if the cotton is not removed it will stay in this condition. Each tube contains enough silk for a laparotomy. The silk and needles are kept during the operation in sterilized water at a temperature of 212 degrees. This may not be necessary, but if the cotton has been partially displaced from the tube, it would be a wise precaution.

In the same boiling water I keep the small glass drainage and the large irrigation tubes. As our hydrant water is generally muddy, I use sterilized water, and always have it boiled in vessels kept for this special purpose. The instruments are washed with sapolio or some strong soap, and boiling water is poured over them, when I begin the operation. The hairs of the brush are pushed through the eyes of the needles and the holes in the instruments so as to get away all poisonous matter. It is well to have instruments, towels, dressings, etc., sterilized for an hour before using them, but they should be thoroughly washed before sterilization.

The patient is given one or more hot baths by a well-trained nurse; the vagina and rectum are washed with copious injections of hot water, and the pubes is shaved. Before making the abdominal incision the abdomen is again washed with soap and brush, and wiped off with sulphuric ether. Dry towels, covered by towels wrung out of boiling water are placed over the abdomen so as to prevent anything possibly unclean coming in contact with the hands or any of the appliances used. The nails are closely cut and the hands thoroughly washed with brush and soap before the operation.

The nurses in charge of the sponges, needles and sutures are as aseptic as the operator. I use no antiseptic solutions, but use for sponges, instruments and hands, boiled sterilized water kept as hot as can be borne. If everything is aseptic we don't need antiseptics; and they may cause general or local trouble. I will refer to but a few points in the technique of the operation. Adhesions are carefully separated close to the tumor or structure to be removed, or the uterus, to prevent hemorrhage or wounding the intestines or bladder. Adherent intestines should be separated if possible, otherwise the operation is incomplete, and the patient will not probably be permanently, if at all, relieved.

The patients sometimes suffer more after the operation than before it, because of the extensive adhesions induced by uncleanliness, antiseptics, or traumatism committed by a careless operator. I believe adhesions will be fewer if antiseptics are absolutely excluded from the operating room, and are not even used for the instruments or the hands. This may seem heterodoxical to many, but I have arrived at this conclusion after experience and careful observation. If the instruments and the hands are clean we need no antiseptics, and if they are unclean the solutions will not cleanse them, or prevent infection, but may so irritate the peritoneum as to cause few or many adhesions. It will require more experience to decide how much damage is done in this way. Blood, pus and all foreign matter should be removed, and great care should be practiced to prevent rupturing a pus sac or cavity in an operation for their removal.

When any foreign matter except blood has gotten into the cavity it should be thoroughly irrigated with hot sterilized water.

This is not only the best way to cleanse the peritoneum, but it is also an excellent means of preventing or treating shock. This may be done by attaching one end of a three-foot piece of gum hose to a glass tube, and the other end to an iron-granite funnel, into which water is copiously poured and forced by hydraulic pressure through all parts of the abdomen and pelvis.

The drainage tube is sometimes invaluable, but if improperly used it is capable of doing much mischief. There are many cases in which it is indicated; there are many in which it is not. It should be used if we close the abdomen before hemorrhage has ceased, or if foreign matter, that is possibly septic, has gotten into the abdomen. It should be attentively cared for and frequently emptied with a long nozzle syringe by a well trained nurse. It should be very small and light, with open end, and numerous fine openings on the sides. It should be carefully placed and long enough to enter to the deepest part of the pelvis.

After the dressings are applied around the tube, a twelve inch square piece of gum dam, with a small hole cut in the center, should be closely fitted around the neck so as to keep the dressings clean. If a piece of absorbent cotton is kept over the mouth of the tube and held in position by folding over the gum cloth, it will absorb discharges and remove the danger of sepsis from the introduction of pathogenic germs. It should be removed when soiled and a new piece used.

Some of our best known laparotomists use too large drainage tubes and do not protect the dressings and the wound by the gum dam. A small tube will usually drain as well as a large one, and it does not subject the patient to so many dangers.

While it has been shown by Grawitz that the peritoneum may render harmless, and dispose of pus or pathogenic germs, it would be reckless to expect it to do so when we may supplant the efforts of nature by the use of a drainage tube through which irrigations may be used if needed. The long nozzle syringe, or a syringe with a small gum tubing attached, affords the best means of emptying the tube, and this can be done aseptically. The practice of trying to drain the peritoneal cavity by introducing strips of gauze or wick into the tube to its bottom, or allowing shreds to enter the cavity as practiced by German laparotomists, is bad surgery, and may be a means of introducing septic matter. While aseptic gauze may usually drain efficiently, it sometimes prevents drainage and causes the blood to coagulate in the tube. This is especially true where capillary drainage is attempted by the use of the wick. I have never seen coagulation where the syringe was used. Probably the most correct exposition of the methods of drainage in Germany will be found in the paper, "Drainage in Laparotomy," by Saenger, of Leipsic, at the recent meeting of the Tenth International Medical Congress at Berlin. No mention is made of protecting the dressings from the discharges by the use of gum dam, or of removing the secretions with the syringe.

Vaginal drainage, with possibly a few exceptions, should never be attempted, though Dr. August Martin, of Berlin, and other German operators frequently practice it. It can accomplish nothing more than supra-pubic drainage and subjects the patient to greater dangers from sepsis. The tube should be removed as soon as the conditions will admit, and when bleeding has practically ceased, and there is only a small quantity of clear inodorous liquid removed, it is no longer needed. If the tube has to be retained more than forty-eight hours, it should be rotated a little twice daily so as to facilitate drainage by preventing obstruction in the small openings.

The dressings need not be disturbed to remove the tube, and in a few weeks the place where it was introduced can scarcely be detected; and ventral hernia will not occur at this point more easily than at any other part of the incision. Hernia in any case will seldom occur if we are careful to unite the ends of the abdominal fascia. This may be done by the deep suture if the fascia is drawn out and the needle correctly introduced, but the separate suturing of the fascia is more reliable.

Recognizing the fact that in laparotomy work death is too often caused by septic infection, and that this can nearly always be prevented, I am deeply in earnest in my desire to aid in impressing upon the medical profession what I conceive to be the best means of preventing the introduction of septic matter. As death occasionally follows prolonged anesthesia in organic diseases of the heart, lungs or kidneys, we should carefully examine these organs before we decide to operate.

